# Bilateral Simultaneous Patellae Fractures Following Knee Trauma: A Case Report

**DOI:** 10.7759/cureus.105751

**Published:** 2026-03-24

**Authors:** Collen Nkosi, Bulelwa Klaas

**Affiliations:** 1 Orthopaedic Surgery, University of the Witwatersrand, Johannesburg, Johannesburg, ZAF

**Keywords:** extensor mechanism, fractures, knee, patella, patellae

## Abstract

Bilateral simultaneous fractures of the patellae were first documented in the literature in the early 19th century, and they remain uncommon today. The patella is the largest sesamoid bone in the body and plays an important role in the knee extensor mechanism. Isolated bilateral patellar fractures following trauma are exceedingly rare (2-3% of all patellar fractures) and most commonly occur with dashboard injuries or various pathological disorders. We report a case of a 43-year-old healthy African male who presented with bilateral simultaneous extensor mechanism disruption following knee trauma. Bilateral open reduction and internal fixation were performed. Postoperatively, the patient was immobilised and discharged in a wheelchair, and attained union without complications. He was routinely observed, demonstrating a fair recovery of range of motion. Extensor mechanism disruption includes a vast differential diagnosis, with a thorough history, examination, and investigations being key elements in confirming a diagnosis. In adults, these injuries often do not result in excellent outcomes.

## Introduction

The extensor mechanism of the knee is a complex system primarily composed of the quadriceps muscle, quadriceps tendon, patella, patellar retinaculum, patellar tendon, surrounding soft tissues, and tibial tuberosity [1,2]. Its interruption can occur due to either a fracture or a tendon rupture, leading to an inability to achieve and maintain knee extension [2]. Bilateral simultaneous fractures of the patellae are extremely uncommon, accounting for less than 3% of all fractures in this anatomical region. They usually result from stress fractures, direct trauma, or pathological fractures [3,4]. Fractures of the patellae in the extensor mechanism complex have been reported to occur six times more often than injuries to the patellar and quadriceps tendons [2]. Bilateral simultaneous patellar fractures are common in patients aged 15 to 65 years, with a higher prevalence in males than in females, and the most frequently observed fracture type is transverse [5]. We discuss a case of a 43-year-old otherwise healthy African male who presented with bilateral simultaneous extensor mechanism disruption after trauma to the patellae.

## Case presentation

**Figure 1:**
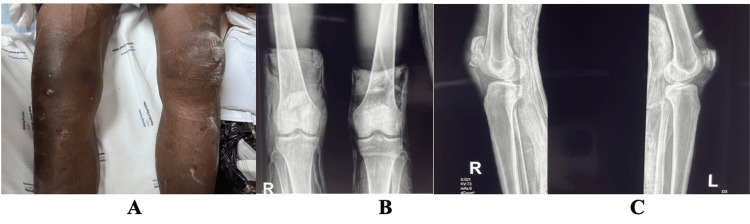
Clinical image and radiographs A: a clinical picture of the superficial abrasion on the legs. B and C: radiographs of the bilateral patellae - anteroposterior and lateral views showing fracture of the patellae

A 43-year-old male patient presented to our casualty department with general body pain, bilateral severe knee pain, and an inability to stand or walk after being allegedly assaulted by a mob for hitting someone in the community. He had been brought in by paramedic personnel on a stretcher. He claimed to have been hit directly on the knees by a digging stick or hoe handle. The orthopaedic and trauma registrars on call cleared him according to Advanced Trauma Life Support protocols and ruled out the possibility of a crush injury. He denied any previous medical history or use of medications. He had superficial abrasions on both of his lower limbs, as well as tram track lines on his lower limbs. Both knees were swollen, had anterior tenderness, and tested positive for knee effusion (Figure 1). On movement, he was unable to extend both knees or maintain extension. His neurovascular status was intact. A clinical diagnosis of bilateral extensor mechanism disruption was made.

Knee X-rays were ordered and revealed bilateral fractures of the patella. The right patella showed a comminuted mid-patellar fracture, whereas the left patella demonstrated a comminuted superior pole fracture (Figure 1). In the emergency department, he was cleaned and immobilised with bilateral above-knee back slabs. Additionally, he received analgesics, a single, immediate 0.5 mL dose of tetanus toxoid, and intravenous fluids.

**Figure 2:**
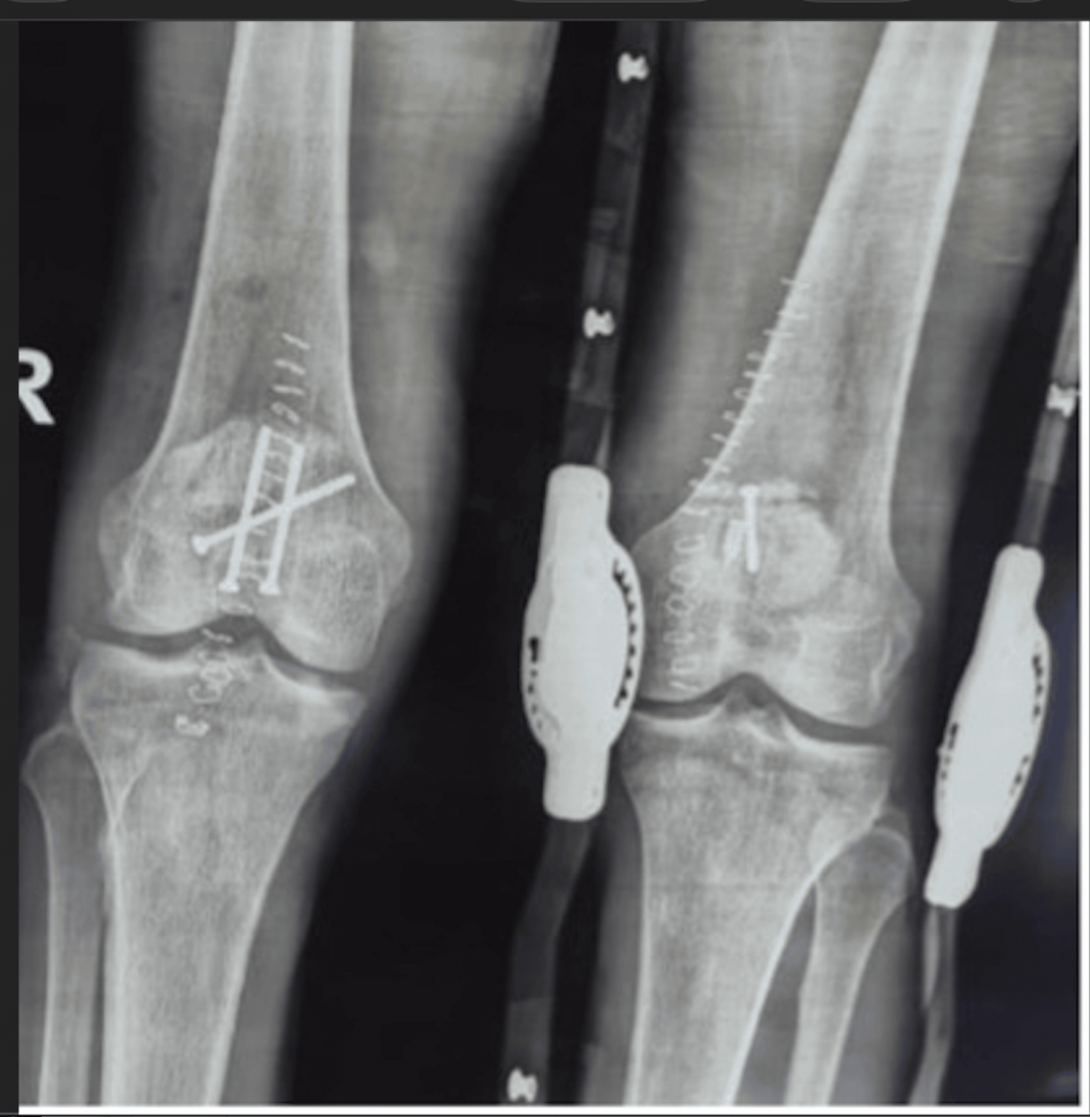
Radiographs of bilateral patellae (anteroposterior views) showing fixed patellar fractures

A surgical procedure was thoroughly discussed with the patient, taking into account the severity of his knee injuries. On day one after admission, the patient was taken to the operating theatre for open reduction and internal fixation. On the right patella, three cannulated screws augmented with fibre tape were used for fixation, and on the left patella, a screw and suture anchor augmented with fibre tape were used for fixation (Figure 2).

**Figure 3:**
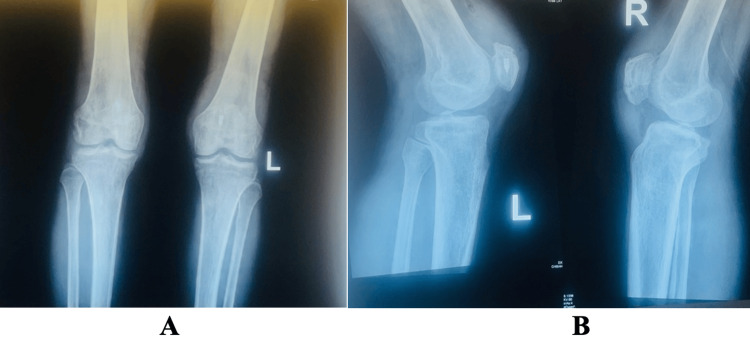
Radiographs of bilateral patellae (anteroposterior and lateral views) demonstrating healed patellae

Postoperatively, the patient was fitted with bilateral knee braces set at a 0-30° range of motion. Three doses of Kefzol were administered, and the patient was discharged 24 hours later in a wheelchair provided by the occupational therapist. The skin clips were removed after two weeks of follow-up, and the physiotherapy team increased the range of motion from 30 to 60 degrees; at four weeks, it was 60 to 90 degrees; and at six weeks, the range-of-motion brace was removed. He continued to see the physiotherapist for six months after surgery, and he had a comparable range of motion with healed fractures and symptomatic implants, which were subsequently removed (Figure 3). Following the removal of implants at eight weeks, the patient's Knee Injury and Osteoarthritis Outcome Score (KOOS) [6] was 47% (symptoms and stiffness: 61%; pain: 47%; function in daily living: 47%; function in sports and recreational activities: 35%; quality of life: 44%).

## Discussion

The extensor mechanism of the knee includes the largest sesamoid bone in the body, called the patella. The patella is situated within the tendon of the quadriceps femoris and serves as the key structure in knee extension. It functions as a lever arm within the knee extensor mechanism, enhancing the effectiveness of the quadriceps [2,7,8]. Traumatic bilateral simultaneous patellar fractures are secondary to direct or indirect trauma to the anterior knee, compressive forces on the extensor mechanism of the knee, or repetitive stress to the patellae [5]. Indirect fractures frequently occur as a result of a rapid contraction of the quadriceps muscles when the knees are flexed, resulting in transverse fracture patterns [9]. Our patient sustained direct trauma to the anterior knees. Desault reported a male patient with bilateral simultaneous patellar fractures in his 1817 paper, which is regarded as the first of its kind [10]. Bilateral simultaneous fractures of the patellae are uncommon, and there has never been a significant review since Steinke's publication in 1913, which included 44 cases [5]. 

Recently, bilateral simultaneous patellar fractures were reported in a 27-year-old male patient following a motor vehicle accident with a dashboard injury. He was treated with open reduction and internal fixation using traditional fixation with Kirschner wires, a cost-effective method, and had good clinical outcomes [11]. Heitzmann et al. reported a case of a 51-year-old female amateur runner who had atraumatic bilateral patellar fractures, which were treated with open reduction and internal fixation using a hybrid approach of screws and tension band wire, with positive outcomes postoperatively [12]. In our case, a hybrid approach was used to treat the fractures, and our patient had fair clinical outcomes.

Our search of the relevant literature in English did not reveal any studies that reported on treatment and functional outcomes using patient-reported outcome measures following bilateral simultaneous patellar fractures, highlighting the rarity of this type of injury. LeBrun et al. analysed the intermediate functional outcomes of 40 patients who underwent surgical treatment for isolated patella fractures. Their study followed these patients for an average of 6.5 years. Their patients had diverse surgical interventions, such as tension band wires, hybrid techniques (screws and tension band wires), wire cerclage, and partial patellectomy. According to their research, patella fractures typically have poor outcomes [13]. However, our patient had better functional results.

## Conclusions

This case report emphasises the importance of maintaining a high index of suspicion and careful clinical and radiographic examinations for trauma patients presenting with bilateral knee pain. Trauma is the most common injury mechanism in patients with bilateral simultaneous patellar fractures. Treatment of bilateral simultaneous patellar fractures is challenging due to delayed mobilisation, quadriceps muscle weakness, and the nature of the injuries. To improve the quality of life in these patients, it is necessary to implement more intensive physiotherapy sessions.
